# Positive nasal culture of methicillin-resistant *Staphylococcus aureus* (MRSA) is a risk factor for surgical site infection in orthopedics

**DOI:** 10.3109/17453670903110675

**Published:** 2009-08-01

**Authors:** Koichi Yano, Yukihide Minoda, Akira Sakawa, Yoshihiro Kuwano, Kyoko Kondo, Wakaba Fukushima, Koichi Tada

**Affiliations:** ^1^Department of Orthopaedic Surgery, Kansai Rosai HospitalHyogoJapan; ^2^Department of Public Health, Osaka City University Graduate School of MedicineOsakaJapan

## Abstract

**Background** Although nasal carriage of MRSA has been identified as one of the risk factors for surgical site infection (SSI) with MRSA, there have been no reports of this in the orthopedics field.

**Methods** This prospective observational cohort study included 2,423 consecutive patients who were admitted to our department over 26 months and who underwent orthopedic surgery. We examined the relationship between pre-existing nasal MRSA and subsequent occurrence of SSI with MRSA.

**Results** 63 patients (2.6%) had a positive nasal MRSA culture. 15 patients (0.6%) developed SSI with MRSA. The occurrence of SSI with MRSA in nasal MRSA carriers was significantly higher than that in non-carriers (4 out of 63 (6.3%) vs. 11 out of 2,360 (0.5%); p < 0.001) (adjusted OR: 11; 95% CI: 3–37; p = 0.001).

**Interpretation** We recommend appropriate treatment of patients who are nasal carriers of MRSA before orthopedic surgery.

## Introduction

The first strains of methicillin-resistant Staphylococcus aureus (MRSA) were reported in the United Kingdom in 1961, only 2 years after the introduction of methicillin (Barber 1961). Since then, similar strains have been isolated in other parts of the world (Barrett et al. 1968, Rountree and Beard 1968). Multidrug-resistant strains have been reported with increasing frequency worldwide, and we only have a few drugs that are effective in treating them (Lowy 1998, Moellering 1998).

Infections caused by resistant strains (Kilgus et al. 2002), such as MRSA, can have serious consequences for patients undergoing orthopedic surgery, especially arthroplasties.

Nasal carriage of MRSA has been identified as one of the risk factors for SSI in surgical patients (Mest et al. 1994, Kluytmans et al. 1997, Davis et al. 2004). It has been reported that nasal colonization with MRSA increases the risk of SSI with MRSA for patients treated in surgical intensive care units (Mest et al. 1994), patients admitted to hospital (Davis et al. 2004), and patients who have undergone continuous ambulatory peritoneal dialysis (CAPD) (Lye et al. 1993). There have, however, been no reports on the relationship between carriership of MRSA in the nasal cavity and SSI with MRSA in orthopedics, especially in relation to joint prostheses. We examined the relationship between the existence of MRSA in the nasal cavity preoperatively and the occurrence of SSI with MRSA after orthopedic surgery.

## Patients and methods

From April 1, 2003 to June 30, 2005, we prospectively investigated all 3,294 consecutive patients who were admitted to the Department of Orthopedic Surgery, which has 140 beds (in a tertiary-care hospital). Of these, 532 patients who underwent non-surgical treatment, 16 patients who had active SSI with MRSA or other strains on admission, and 323 patients who were 17 years of age or younger were excluded. The study group consisted of the remaining 2,423 patients who had orthopedic surgery. Informed consent was obtained from these patients and nasal swabs were taken to check for the presence of MRSA. MRSA carriers were not decolonized before surgery. All patients were followed up for at least 1 year after surgery in order to examine whether they had developed SSI with MRSA. This prospective observational cohort study was approved by the ethical committee of our institution.

### Assessment of nasal MRSA

Both nares were sampled using a swab, at the outpatient clinic for scheduled surgery and at admission for emergency surgery. Swabs samples were cultured aerobically at 35°C for 24–48 h on MRSA I-A agar (Nikken Biomedical Laboratory Co. Ltd., Kyoto, Japan) containing ceftizoxime (25 mg/L) with 4% sodium chloride. Growth of Staphylococcus aureus was confirmed by the Lecithovitellin reaction. Susceptibility of isolates was determined with oxacillin by the dilution method using the Micro Scan system (Dade Behring Inc., West Sacramento, CA) at a minimal inhibitory concentration of < 4 mg/L as breakpoint.

### Patient characteristics

Diagnosis of SSI was based on the criteria put forward by the Centers for Disease Control and Prevention (CDC) (Mangram et al. 1999), and it was classified into 3 groups: (1) superficial incisional surgical-site infection occurring within 30 days of surgery, (2) deep incisional surgical-site infection, and (3) organ/space surgical-site infections occurring within 30 days of surgery if no implant was left in place or within 1 year if the implant was in place and the infection appeared to be related to the surgery. Pathogens were examined in all the SSI cases. SSI with MRSA was assessed using the same culture method as used for nasal MRSA.

As risk factors for SSI with MRSA, the following factors were checked: sex, age at operation, history of diabetes mellitus and rheumatoid arthritis, the site of operation, use of a prosthesis, length of the surgical procedure, the existence of open fracture(s), Anaesthesiology Society of America (ASA) physical status classification (Anonymous 1963), and body mass index (BMI). Patients were considered to have diabetes mellitus or active rheumatoid arthritis if they had been treated with any anti-diabetic or anti-rheumatoid (including steroids and/or immunosuppressive) drugs. The site of operation was divided into three groups: upper extremity (from clavicle to finger), spine (from cervical spine to lumbar spine), or lower extremity (from pelvis and hip to toe). The use of a prosthesis for knee and hip, including total hip arthroplasty and femoral head prosthetic replacement, was defined as use of a prosthesis. The ASA classification involves 5 scores (from 1 to 5); the higher the score, the worse the general condition (Anonymous 1963).

### Statistics

Patients admitted several times during the study period were included only once in the analysis. ASA classes 3 and 4 were combined because the number of patients with class 4 was small, and no patients were classified as class 5. Thus, ASA score was divided into 3 groups. Differences between the groups were assessed by Chi square test or Wilcoxon rank sum test. Fisher's exact test was also used where appropriate. Multiple logistic regression analysis was performed to estimate adjusted odds ratios (ORs) and their 95% confidence intervals (CIs). The test for trend was performed by including explanatory variables in the model that were coded by ordinal numbers with increasing categories of exposure. All statistical analyses were performed using SAS software version 9.1 (SAS Institute, Inc., Cary, NC). Statistical significance was set at p < 0.05.

## Results

2,423 patients (1,272 males and 1151 females) were studied. The average age at admission was 55 years (SD 19; range 18–96). The average length of the surgical procedure was 117 min (SD 85; range 5–935). 1,059 patients were classified as ASA class 1, 1,079 patients as class 2, 280 patients as class 3, and 5 patients as class 4. The average BMI on admission was 23 (SD 3.9; range 15–36). The average follow-up period was 26 (13–38) months. Of these patients, 63 (2.6%) had preoperative nasal cultures that were positive for MRSA. Overall, 15 patients (0.6%) developed SSI with MRSA postoperatively. The mean time of onset of SSI with MRSA was 1.1 months (range 4 days to 3 months). No other organisms were detected in these 15 patients. According to the criteria defined by CDC, 6 patients had superficial incisional SSI, 1 patient had deep incisional SSI, and 8 patients had organ/space SSI. 1 patient had removal of a joint prosthesis, and 2 patients had limb amputation (Table 1). 3 patients with joint prostheses (1 femoral head prosthesis and 2 total knee prostheses) had SSI with MRSA. The latter 2 patients who had total knee replacement could retain their prostheses by debridement and antibiotic treatment. For the 1 patient who had replacement of a femoral head prosthesis, debridement and antibiotic treatment were not effective and removal of prosthesis was required.

**Table 1.** Demographic and clinical data of 16 patients with MRSA surgical site infection.

No.	Culture of nasal MRSA	Site of **^a^** operation	Sex **^b^**	Age	Usage of prosthesis	Infection onset after the operation **^c^**	Criteria of SSI **^d^**	Number of additional operations	Outcome
1	+	L	M	56	–	4W	S	1	Resolved
2	+	S	F	75	–	3M	O/S	1	Resolved
3	+	S	F	69	–	2W	O/S	2	Resolved
4	+	L	F	68	–	4W	D	2	Amputated
5	–	U	M	58	–	4W	S	1	Resolved
6	–	L	M	70	+	4W	O/S	1	Resolved
7	–	U	F	54	–	2W	S	2	Resolved
8	–	L	F	60	+	1M	S	1	Resolved
9	–	L	M	74	–	1M	S	1	Resolved
10	–	L	M	21	–	3W	S	1	Resolved
11	–	U	M	58	–	4D	O/S	1	Resolved
12	–	L	M	44	–	3M	O/S	3	Resolved
13	–	L	M	50	–	3W	O/S	2	Amputated
14	–	L	F	81	+	2W	O/S	4	Removed
15	–	L	M	70	–	4W	O/S	1	Resolved

^a^ U; upper extremity, S; spine, L; lower extremity

^b^ M; male, F; female

^c^ D; day, W; week, M; month

^d^ S; superficial, D; deep, O/S; organ/space

Patients with preoperative nasal cultures that were positive for MRSA had a higher occurrence of SSI with MRSA (6.3%) than patients who were negative for nasal MRSA preoperatively (0.5%), by crude rate analysis (p < 0.001) (Table 2). Patients with nasal cultures that were positive for MRSA preoperatively were statistically significantly older than patients who were negative for MRSA in nasal cultures, both in the total group (median age: 69 vs. 59, p = 0.001) and in the SSI negative group (median age: 69 vs. 59, p = 0.001). However, in the SSI-positive group, the difference in age between patients who were positive or negative for MRSA in nasal swabs preoperatively was not statistically significant (median age: 69 vs. 59, p = 0.5). There was no significant association between gender and preoperative nasal culture status in any of the 3 groups.

**Table 2.** Crude and adjusted ORs and 95%CIs for surgical site infection with MRSA using logistic regression model.

Variable		Surgical site infection		Crude		Adjusted ^a^	
		n / N	(%)	OR (95% CI)	p-value	OR (95% CI)	p-value
Culture of nasal MRSA	–	11 / 2360	(0.5)	1		1	
	+	4 / 63	(6.3)	15 (4.5–47)	<0.001	11 (3.0–37)	<0.001
Sex	M	9 / 1272	(0.7)	1		1	
	F	6 / 1151	(0.5)	1.4 (0.5–3.8)	0.6	1.7 (0.5–5.4)	0.4
Age per 10-year increase				1.2 (0.9–1.6)	0.3	1.1 (0.7–1.5)	0.8
Length of surgical procedure per 10-minute increase				1.0 (1.0–1.1)	0.1	1.0 (1.0–1.1)	0.2
ASA class	1	3 / 1059	(0.3)	1		1	
	2	7 / 1079	(0.7)	2.3 (0.6–8.9)	0.2	2.2 (0.5–10)	0.3
	3, 4	5 / 285	(1.8)	6.3 (1.5–27)	0.01	4.2 (0.6–27)	0.1
Trend				p=0.04		p=0.1	
BMI			1.0 (0.9–1.1)	0.7	1.0 (0.9–1.1)	0.8	

^a^ model included usage of prosthesis, site of infection, open fracture, history of diabetes mellitus, and history of rheumatoid arthritis, and all variables in the table.

Possible associations between SSI with MRSA and perioperative factors (sex, age, history of diabetes mellitus, history of rheumatoid arthritis, the site of operation, use of a prosthesis, length of the surgical procedure, the existence of open fracture(s), ASA class, and BMI), as well as preoperative culture of nasal MRSA, were examined. By crude rate analysis, sex, age, history of diabetes mellitus and rheumatoid arthritis, the site of operation, use of a prosthesis, length of the surgical procedure, existence of open fracture(s), an ASA class of 1 or 2, and BMI were not statistically significant factors for SSI with MRSA. Nasal cultures that were positive for MRSA preoperatively and an ASA class of 3 or 4 were associated with SSI with MRSA by crude rate analysis (OR: 15; 95% CI: 4.5–47; p < 0.001; and OR: 6, 95% CI: 1.5–27; p = 0.01, respectively). Multivariate logistic regression analysis showed that preoperative culture of nasal MRSA was only statistically significantly associated with SSI with MRSA even after controlling for perioperative factors (adjusted OR: 11; 95% CI: 3–37; p < 0.001). Thus, positive culture of nasal MRSA independently increased the risk of SSI with MRSA by 11 times (Table 2).

Concerning the 4 patients who had positive results of culture of nasal MRSA preoperatively and SSI with MRSA subsequently, the susceptibility patterns of the MRSA isolates obtained from the nasal cavity and from the surgical site were the same.

## Discussion

We found that the adjusted OR of nasal MRSA for SSI with MRSA after orthopedic surgery was 11 after controlling for the effects of potential confounders. Mest et al. (1994) reported a prospective study on patients who were treated in the surgical intensive care unit from all surgical wards. They screened all patients preoperatively for nasal carriage of MRSA and found that 4% of patients had MRSA-positive nasal cultures. 26% of these MRSA carriers developed MRSA infections, as compared to 1.3% of those who were not carriers. Davis et al. (2004) reported a prospective study on subsequent MRSA infections in patients admitted to 5 units in their hospital. 3% of the patients were MRSA carriers. The rate of subsequent MRSA infections in nasal carriers of MRSA was 10 times as much as that in non-carriers. Lye et al. (1993) reported a prospective study on MRSA infection in patients entering into the continuous ambulatory peritoneal dialysis (CAPD) program. 17% of these patients were nasal carriers of MRSA. Nasal carriage was associated with a statistically significant increase in the rate of postoperative MRSA infection, such as peritonitis, catheter losses, and dropouts of CAPD (Lye et al. 1993). Our findings in orthopedic surgery were similar to these previous reports regarding the relationship between nasal carriership of MRSA and MRSA infection. However, the rates of nasal carriership of MRSA and MRSA infections were lower in our study. The difference may have been due to differences in the condition of patients, and differences in procedures.

The length of the surgical procedure, history of diabetes mellitus and rheumatoid arthritis, ASA class, and BMI have been reported to be risk factors for SSI caused by organisms other than MRSA in general surgery, orthopedic surgery, cardiovascular surgery, and neurosurgery (Gil-Egea et al. 1987, Nagachinta et al. 1987, Wymenga et al. 1992, Pons et al. 1993, Slaughter et al. 1993, He et al. 1994, Barber et al. 1995, Ziv et al. 1996, Flynn et al. 2000, Syahrizal et al. 2001, Dindo et al. 2003). However, there have been no reports on the relationship between such factors and SSI with MRSA. Apart from nasal MRSA, we thus used these as confounding factors in the multivariate logistic regression analysis. ASA class 3 and 4 was significantly associated with SSI with MRSA in crude analysis. However, it was not significant in the adjusted analysis. This may have been because number of patients with higher ASA scores was small in this study.

In some reports, nasal mupirocin ointment was found to reduce the risk of *S. aureus* infection (Reagan et al. 1991, Kluytmans et al. 1995, 1996, Gernaat-van der Sluis et al. 1998), but this was not found to be the case in other reports (Kalmeijer et al. 2002, Perl et al. 2002). Recently, combination therapy with nasal mupirocin ointment and washing with disinfectants was reported to reduce MRSA infection after surgery (Wilcox et al. 2003, Simor et al. 2007).

One weakness of our study was that although we found exactly the same susceptibility patterns for MRSA isolates obtained from the nasal cavity and for those in surgical-site infections, we did not perform phage typing. Phage typing could be used in future studies to show whether the SSI is caused by the same strain of MRSA as that cultured from the nasal cavity.

We believe that our findings are of clinical importance. We recommend preoperative nasal culture for MRSA and, if possible, appropriate treatment for nasal carriers of MRSA.

## References

[CIT0001] Anonymous (1963). New classification of physical status.. Anesthesiology.

[CIT0002] Barber M (1961). Methicillin-resistant staphylococci.. J Clin Pathol.

[CIT0003] Barber GR, Miransky J, Brown AE, Coit DG, Lewis FM, Thaler HT, Kiehn TE, Armstrong D (1995). Direct observations of surgical wound infections at a comprehensive cancer center.. Arch Surg.

[CIT0004] Barrett FF, McGehee RF, Finland M (1968). Methicillin-resistant Staphylococcus aureus at Boston City Hospital. Bacteriologic and epidemiologic observations.. N Engl J Med.

[CIT0005] Davis KA, Stewart JJ, Crouch HK, Florez CE, Hospenthal DR (2004). Methicillin-resistant Staphylococcus aureus (MRSA) nares colonization at hospital admission and its effect on subsequent MRSA infection.. Clin Infect Dis.

[CIT0006] Dindo D, Muller MK, Weber M, Clavien PA (2003). Obesity in general elective surgery.. Lancet.

[CIT0007] Flynn JM, Rodriguez-del Rio F, Piza PA (2000). Closed ankle fractures in the diabetic patient.. Foot Ankle Int.

[CIT0008] Gernaat-van der Sluis AJ, Hoogenboom-Verdegaal AM, Edixhoven PJ, Spies-van Rooijen NH (1998). Prophylactic mupirocin could reduce orthopedic wound infections. 1,044 patients treated with mupirocin compared with 1,260 historical controls.. Acta Orthop Scand.

[CIT0009] Gil-Egea MJ, Pi-Sunyer MT, Verdaguer A, Sanz F, Sitges-Serra A, Eleizegui LT (1987). Surgical wound infections: prospective study of 4,468 clean wounds.. Infect Control.

[CIT0010] He GW, Ryan WH, Acuff TE, Bowman RT, Douthit MB, Yang CQ, Mack MJ (1994). Risk factors for operative mortality and sternal wound infection in bilateral internal mammary artery grafting.. J Thorac Cardiovasc Surg.

[CIT0011] Kalmeijer MD, Coertjens H, van Nieuwland-Bollen PM, Bogaers-Hofman D, de Baere GA, Stuurman A, van Belkum A, Kluytmans JA (2002). Surgical site infections in orthopedic surgery: the effect of mupirocin nasal ointment in a double-blind, randomized, placebo-controlled study.. Clin Infect Dis.

[CIT0012] Kilgus DJ, Howe DJ, Strang A (2002). Results of periprosthetic hip and knee infections caused by resistant bacteria.. Clin Orthop Relat Res.

[CIT0013] Kluytmans JA, Mouton JW, Ijzerman EP, Vandenbroucke-Grauls CM, Maat AW, Wagenvoort JH, Verbrugh HA (1995). Nasal carriage of Staphylococcus aureus as a major risk factor for wound infections after cardiac surgery.. J Infect Dis.

[CIT0014] Kluytmans JA, Mouton JW, VandenBergh MF, Manders MJ, Maat AP, Wagenvoort JH, Michel MF, Verbrugh HA (1996). Reduction of surgical-site infections in cardiothoracic surgery by elimination of nasal carriage of Staphylococcus aureus.. Infect Control Hosp Epidemiol.

[CIT0015] Kluytmans J, Belkum A, Verbrugh H (1997). Nasal carriage of Staphylococcus aureus: epidemiology, underlying mechanisms, and associated risks.. Clin Microbiol Rev.

[CIT0016] Lowy FD (1998). Staphylococcus aureus infections.. N Engl J Med.

[CIT0017] Lye WC, Leong SO, Lee EJ (1993). Methicillin-resistant Staphylococcus aureus nasal carriage and infections in CAPD.. Kidney Int.

[CIT0018] Mangram AJ, Horan TC, Pearson ML, Silver LC, Jarvis WR (1999). Guideline for prevention of surgical site infection, 1999. Hospital Infection Control Practices Advisory Committee.. Infect Control Hosp Epidemiol.

[CIT0019] Mest DR, Wong DH, Shimoda KJ, Mulligan ME, Wilson SE (1994). Nasal colonization with methicillin-resistant Staphylococcus aureus on admission to the surgical intensive care unit increases the risk of infection.. Anesth Analg.

[CIT0020] Moellering RC (1998). Problems with antimicrobial resistance in gram-positive cocci.. Clin Infect Dis.

[CIT0021] Nagachinta T, Stephens M, Reitz B, Polk BF (1987). Risk factors for surgical-wound infection following cardiac surgery.. J Infect Dis.

[CIT0022] Perl TM, Cullen JJ, Wenzel RP, Zimmerman MB, Pfaller MA, Sheppard D, Twombley J, French PP, Herwaldt LA (2002). Intranasal mupirocin to prevent postoperative Staphylococcus aureus infections.. N Engl J Med.

[CIT0023] Pons VG, Denlinger SL, Guglielmo BJ, Octavio J, Flaherty J, Derish PA, Wilson CB (1993). Ceftizoxime versus vancomycin and gentamicin in neurosurgical prophylaxis: a randomized, prospective, blinded clinical study.. Neurosurgery.

[CIT0024] Reagan DR, Doebbeling BN, Pfaller MA, Sheetz CT, Houston AK, Hollis RJ, Wenzel RP (1991). Elimination of coincident Staphylococcus aureus nasal and hand carriage with intranasal application of mupirocin calcium ointment.. Ann Intern Med.

[CIT0025] Rountree PM, Beard MA (1968). Hospital strains of Staphylococcus aureus, with particular reference to methicillin-resistant strains.. Med J Aust.

[CIT0026] Simor AE, Phillips E, McGeer A, Konvalinka A, Loeb M, Devlin HR, Kiss A (2007). Randomized controlled trial of chlorhexidine gluconate for washing, intranasal mupirocin, and rifampin and doxycycline versus no treatment for the eradication of methicillin-resistant Staphylococcus aureus colonization.. Clin Infect Dis.

[CIT0027] Slaughter MS, Olson MM, Lee JT, Ward HB (1993). A fifteen-year wound surveillance study after coronary artery bypass.. Ann Thorac Surg.

[CIT0028] Syahrizal AB, Kareem BA, Anbanadan S, Harwant S (2001). Risk factors for infection in total knee replacement surgery at hospital Kuala Lumpur.. Med J Malaysia (Suppl D).

[CIT0029] Wilcox MH, Hall J, Pike H, Templeton PA, Fawley WN, Parnell P, Verity P (2003). Use of perioperative mupirocin to prevent methicillin-resistant Staphylococcus aureus (MRSA) orthopaedic surgical site infections.. J Hosp Infect.

[CIT0030] Wymenga AB, van Horn JR, Theeuwes A, Muytjens HL, Slooff TJ (1992). Perioperative factors associated with septic arthritis after arthroplasty. Prospective multicenter study of 362 knee and 2,651 hip operations.. Acta Orthop Scand.

[CIT0031] Ziv Y, Church JM, Fazio VW, King TM, Lavery IC (1996). Effect of systemic steroids on ileal pouch-anal anastomosis in patients with ulcerative colitis.. Dis Colon Rectum.

